# Network Association of Biochemical and Inflammatory Abnormalities With Psychiatric Symptoms in First-Episode Schizophrenia Patients

**DOI:** 10.3389/fpsyt.2022.834539

**Published:** 2022-02-22

**Authors:** Junwei Yan, Yuanyuan Chen, Peijun Ju, Jianliang Gao, Loufeng Zhang, Jingwei Li, Keming Wang, Jie Zhang, Chao Li, Qingrong Xia, Cuizhen Zhu, Xulai Zhang

**Affiliations:** ^1^Affiliated Psychological Hospital of Anhui Medical University, Hefei, China; ^2^Department of Medical Education and Research, Hefei Fourth People's Hospital, Hefei, China; ^3^Department of Medical Education and Research, Anhui Mental Health Center, Hefei, China; ^4^Department of Medical Education and Research, Anhui Clinical Center for Mental and Psychological Diseases, Hefei, China; ^5^Department of Psychiatry, Shanghai Mental Health Center, Shanghai, China; ^6^Shanghai Jiao Tong University School of Medicine, Shanghai, China; ^7^Department of Psychiatry, Shanghai Key Laboratory of Psychotic Disorders, Shanghai, China

**Keywords:** biochemical index, cardiovascular risk factors, electrochemiluminescent immunoassay, inflammation cytokine, schizophrenia

## Abstract

**Background:**

Cardiovascular disease (CVD) risk factors such as dyslipidemia and systemic aberrant inflammatory processes may occur in patients with psychotic disorders, which may cause increased mortality. The interplay between immune and metabolic markers and its contribution to the clinical symptoms of schizophrenia (SCZ) remain unclear. This study aimed to examine the association of a series of inflammatory factors, plasma biochemical indicators, and SCZ clinical symptomatology with the severity of SCZ symptoms.

**Methods:**

A total of 115 participants, including 79 first-episode drug-naïve patients with SCZ and 36 healthy controls, were enrolled in this study. Semi-structured interviews were used to collect sociodemographic data, family history of SCZ, and medical and psychiatric history. The Brief Psychiatric Rating Scale (BPRS) and the Positive and Negative Syndrome Scale (PANSS) were administered by a clinical psychiatrist to evaluate the symptom severity of patients with SCZ. Plasma inflammatory cytokines were measured by a fully automated electrochemiluminescent immunoassay (Meso Scale Discovery).

**Results:**

Blood routine, biochemical, and inflammation cytokine test results showed that the levels of white blood cell count, neutrophil count, natrium, CRP, IL-8, IL-6, IL-13, and IL-16 significantly increased in the case group than in the healthy controls (*p* < 0.05), whereas levels of red blood cell count, hemoglobin concentration, mean corpuscular hemoglobin concentration, total protein, albumin, total bile acid, high-density lipoprotein (HDL), apolipoprotein A1, blood urea nitrogen, kalium and IL-15 were lower than in the healthy controls (*p* < 0.05). Correlation network analysis results shown that the natrium, HDL and red blood cell count were the top 3 factors closely to with BPRS and PANSS related clinical symptoms among of correlation network (degree = 4). ROC curve analysis explored the IL-16, IL-8, IL-13, IL-15, natrium, and HDL had highly sensitivity and specificity to the predictive validity and effectiveness for SCZ symptoms.

**Conclusion:**

Our study revealed a complex interactive network correlation among the cardiovascular risk factors, biological immunity profiles, and psychotic symptoms in first-episode patients. Abnormal inflammatory factors and CVD risk factors had high sensitivity and specificity for predicting SCZ symptoms. Generally, our study provided novel information on the immune-related mechanisms involved in early CVD risk in patients with psychotic disorders.

## Introduction

Schizophrenia (SCZ) is a chronic mental illness with a lifetime prevalence of nearly 1% of the world population (1). SCZ is now widely acknowledged to contribute substantially to the global burden of disease, belonging to one of the top 10 causes of disability (1, 2). Patients with SCZ are characterized by positive symptoms, negative symptoms, and cognitive deficits (3), such as hallucinations, delusions, disorganized behavior and speech, decreased motivation, diminished expressiveness, impaired executive functions, asociality, avolition, and blunted affect (1, 4). Although the clinical diagnosis of SCZ primarily relies on the immediate symptomatology of patients, evidence now suggests that the biological process underlying the illness has already been ongoing for many years (5, 6), the mechanism of which remains unknown. Distinct categories of risk factors and their internal association may be connected with psychotic symptoms and thus predict a chronic course of SCZ.

Numerous pathophysiological processes have been identified in SCZ, including immune-mediated responses, biochemical metabolism, and white blood cell (WBC) count. These anomalous phenomena can be considered as risk factors for disease progression. Often, psychotic disorders are characterized by significant increased risk of comorbid cardiovascular diseases (CVDs) and a cluster of cardiovascular risk factors, including dyslipidemia, abdominal obesity, hypertension, and hyperglycemia. Convincing evidence shows that abnormal lipid metabolites accumulate together with low-grade inflammation leading to chronic vascular remodeling, which may be the key triggers in the onset and maintenance of SCZ symptoms (7). Accordingly, the immune–inflammatory response system and comorbid CVD risk factors in SCZ have been proposed to play a key role in SCZ pathophysiology. Studies have attempted to prove that inflammatory cytokines may be a risk factor contributing to the exacerbation or alleviation of SCZ symptoms (8). Notably, previous studies have shown heterogeneous features for immune dysfunction in SCZ, leading to difficulty in determining the clear biological markers in this patient group (8, 9). These conditions pose a great challenge to clinicians to use the biological markers as guiding tools in the clinical evaluation of psychosis together with metabolic syndromes.

The observed abnormal profiles of circulating pro-inflammatory cytokines, including Interleukin-1 (IL-1) family, IL-2, IL-4, IL-5, IL-6, IL-8, IL-11, IL-17, IL-18, interferon-γ (IFN-γ), IFN-β, tumor necrosis factor-α (TNF-α), and TNF-β (10, 11), have shown that these disturbed pro-inflammatory cytokines are found to have higher levels in the peripheral blood of patients with first-episode SCZ than in that of healthy controls (12, 13). Some results have suggested an association between pro-inflammatory cytokines and SCZ symptom, particularly negative symptoms (14, 15). A relatively new research direction is the examination of anti-inflammatory cytokines' alterations in SCZ, such as IL-10, IL-12, IL-32, IL-37, and transforming growth factor (16, 17). However, convincing evidence between aberrant expression of inflammatory factors and SCZ symptoms has not yet been obtained (18, 19). The inconsistent results from literature may be explained by the heterogeneity of SCZ (20), and confounding factors need to be considered in future research.

In this study, we hypothesized that these measures of blood routine, biochemical, and inflammatory cytokines were associated with severity of symptoms. Furthermore, the abnormalities of inflammation in patients with SCZ-mediated complex immune–brain interactions, which further mediated the causal link with routine blood test and metabolic risks, eventually contributed to the progression of clinical symptoms of SCZ. Overall, we demonstrated the interactions among many immune network components, various plasma biochemical indicators, and diverse psychosis symptoms.

## Methods

### Ethics Statement

This study was approved by the Medical Ethics Committee of the Anhui Mental Health Center (AMHC). All participants provided written consent prior to study participation in accordance with the principles of the Declaration of Helsinki. The trial clinical registration number was chiCTR1800019343.

### Participants

A total of 147 participants were initially selected, among which 18 participants did not meet the inclusion criteria, nine individuals could not complete the scale assessment, eight people violated protocol, and four patients refused to sign the informed consent. Hence, 32 subjects who did not meet the experimental criteria were excluded from this experiment. The remaining 115 participants were included, and 79 first-episode drug-naïve SCZ patients were enrolled into study groups according to the Diagnostic and Statistical Manual of Mental Disorders, Fifth Edition (DSM-5), All patients were hospitalized at the AMHC between June 2019 and May 2021, and 36 healthy people were recruited by the hospital physical examination center.

According to the trial standards, all participants were assessed using the Mini-International Neuropsychiatric Interview (MINI) 6.0.0. The inclusion criteria for the study group were as follows: (1) fulfillment of the DSM-5 criteria for first-episode state and drug-naïve SCZ by two independent experienced psychiatrists; (2) currently free of allergies, autoimmune diseases, or infections; (3) not taking immunosuppressive or anti-inflammatory drugs; and (4) aged between 16 and 60 years old. The inclusion criteria for the healthy controls were as follows: (1) no personal or family history of mental illness; (2) no current infections, allergies, or autoimmune diseases; (3) no history of any neurological disease or traumatic brain injury; and (4) not taking immunosuppressive or anti-inflammatory drugs. The exclusion criteria were as follows: (1) a history of craniocerebral trauma, organic cerebral diseases, or other mental disorders; (2) a history of alcohol or other substance use; (3) diabetes, hypertension, dyslipidemia, and endocrine disease; (4) pregnant or lactating women; and (5) administration of electroconvulsive therapy without convulsions before enrolment.

### Clinical Assessments

#### Mini-International Neuropsychiatric Interview (MINI) 6.0.0

Participants were screened by two experienced psychiatrists for inclusion in this study. The preliminary clinical diagnosis was verified by MINI 6.0.0, which is a concise diagnostic interview for psychiatric disorders used by psychiatrists in the United States and Europe. All patients underwent MINI 6.0.0 to confirm the clinical diagnosis of first-episode state and drug-naïve SCZ (21).

#### Demographic Characteristics

A self-reported questionnaire was used to collect information concerning gender, age at first episode, duration of disease course, family history, medical conditions, and health-service accessibility.

#### Positive and Negative Syndrome Scale (PANSS)

The PANSS is an extensively used instrument for measuring severe psychopathology in adult patients with SCZ. Versions of the five-factor model have been used in diverse SCZ research areas, including positive symptom, negative symptom, cognitive defect, excited symptom, and depressive symptom (22).

#### Brief Psychiatric Rating Scale (BPRS)

The BPRS was developed as a clinician-rated instrument to assess symptoms of anxiety depression, unusual thought content, uncooperativeness, tension, and hostile suspiciousness in SCZ patients. The 18 items of BPRS were assessed by the rater on a seven-point scale ranging from 1 (absent) to 7 (extremely severe). The total score was calculated by summing the scores from the 18 items, with higher scores indicating more severe psychopathology (23).

### Laboratory Evaluation

Venous blood samples were collected from all participants in the morning after an overnight fast. The time of blood draw for the case group was the day after admission to the hospital. Two samples were sent to the Department of Clinical Laboratory for tests of routine blood count and blood biochemistry within 2 h. One sample was immediately sent to the Department of Clinical Laboratory for centrifugation, and separated plasma was frozen at −80°C until analysis.

Blood routine analysis was performed using an automatic hematology analyzer (Mindray BC-2800, Shenzhen, China). Plasma biochemical parameters were measured with an automatic biochemistry analyzer (AU480, Beckdman Counter, USA) by using commercial kits (Roche, Switzerland). MSD Platform (labservice.univ-bio.com, Shanghai, China) was used to measure multiplex levels of inflammation biomarkers [Flt-1, Placental growth factor (PIGF), Angiopoietin-2 (Tie-2), IFN-γ, TNF-α, TNF-β, C-reactive protein (CRP), IL-1α, IL-2, IL-4, IL-5, IL-6, IL-7, IL-8, IL-10, IL-12, IL-13, IL-15, IL-16, and IL-17A]. MSD sector 2100A was used to read the plates, and data were analyzed using MSD Discovery Workbench 4.0 software (from Meso Scale Diagnostics). The limit of detection for these inflammatory cytokines was 0.01 pg/mL, and all standards and plasma samples were assayed in a duplicate test.

### Statistical Analysis

The Standard Package for Social Sciences software was used for statistical analyses (version 22.0, IBM Corp, Armonk, NY, USA). Values conforming to a normal distribution were expressed as the mean and standard deviation, and conversely, as median and IQR (25%, 75% percentiles). Chi-square tests were applied to categorical data. Student's *t*-tests were applied for continuous data with normal distribution. For data with irregular distribution, the Mann–Whitney *U*-test was used. When multiple comparisons exist, the false discovery rate (FDR) method is used to adjust the *p*-value (24). We adopted partial correlation to assess the correlation between routine blood and biochemical indicators, inflammatory factors, and clinical symptoms, with the control variable of age. Heat and network plots of the bias correlation coefficients of the two variables were filtered using V-search (version v3.5.0) and R (version v3.6.1) software. The area under the receiver-operating characteristic curve was used for the performance assessment of biochemical indicators and inflammatory factors to evaluate the clinical translational value of the factors. Other performance metrics including sensitivity and specificity were also obtained at the optimal cutoff value of 0.5 as defined by the receiver-operating characteristic curve (ROC). All tests were two-tailed, and differences were considered statistically significant at *p* < 0.05.

## Results

### Demographic and Clinical Characteristics of All Subjects

The demographic data of patients and controls are shown in Table 1. We recruited 79 patients with SCZ (54 females and 25 males with a mean age of 34.39 ± 11.85 years) and 36 healthy controls (22 females and 14 males with a mean age of 28.11 ± 5.16 years). The age was higher in the case group than in the healthy controls (*p* = 0.003). No significant differences in sex and BMI were found between the two study groups (*p* > 0.05). The BPRS total scores, PANSS total scores, and scores of the BPRS and PANSS subscales of the SCZ patients are also presented in Table 1.

**Table 1:** Comparison of demographic and clinical data between controls and first-episode drug naïve patients with SCZ.

**Variables**	**Controls**	**SCZ**	***χ*** **^2^/*t* (*P*)**
Sex (female/male)	22/14	54/25	0.579 (0.447)
Age (years)	28.11 ± 5.16	34.39 ± 11.85	3.045 (0.003)
BMI (kg/m^2^)	22.60 ± 2.96	23.46 ± 4.25	1.101 (0.273)
BPRS	-	48.70 ± 9.55	-
Anxiety depression	-	5.51 ± 2.03	-
Thought disturbance	-	11.05 ± 2.88	-
Anergia	-	9.91 ± 2.83	-
Activation	-	4.73 ± 2.13	-
Hostile suspiciousness	-	9.28 ± 3.32	-
PANSS	-	86.91 ± 16.63	-
Positive symptom	-	11.73 ± 3.82	-
Negative symptom	-	20.20 ± 6.27	-
Cognitive defect	-	9.89 ± 2.97	-
Excited symptom	-	11.69 ± 3.95	-
Depressive symptom	-	4.05 ± 1.39	-

*BMI, Body mass index; BPRS, Brief Psychiatric Rating Scale; PANSS, Positive and Negative Syndrome Scale; SCZ, schizophrenia*.

### Results of Blood Routine and Biochemical and Inflammatory Cytokine Concentration

**Figure 1:**
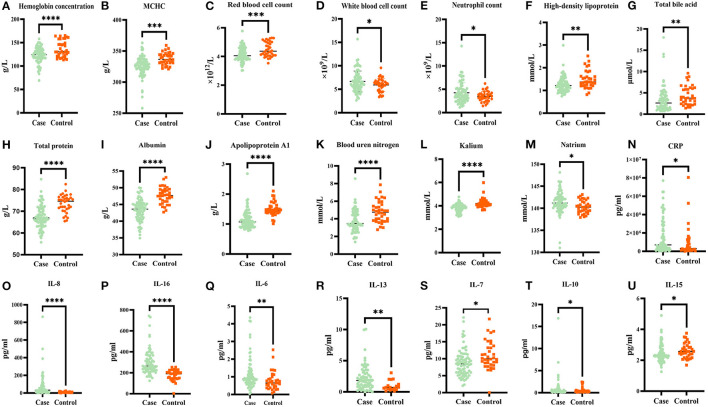
Comparison of blood routine, biochemical factors, and inflammation cytokines concentrations in first episode drug naive patients with schizophrenia and healthy controls. Hemoglobin concentration, mean corpuscular hemoglobin concentration (MCHC), red blood cell count, high-density lipoprotein, total bile acid, total protein, albumin, apolipoprotein A1, blood urea nitrogen, kalium, and IL-7 were lower in the case group than in healthy controls [**(A–C,F–L,S)** *p* < 0.05, respectively]. While white blood cell count, neutrophil count and natrium, CRP, IL-8, IL-16, IL-6, IL-13, IL-10, and IL-15 were higher in case group than in healthy controls [**(D,E,M–R,T,U)** *p* < 0.05, respectively]. The false discovery rate (FDR) method is used to adjust the *p*-value. *p ≤ 0.05, **p ≤ 0.01, ***p ≤ 0.001, ****p ≤ 0.0001.

To investigate the differential internal environment homeostasis between patients and healthy individuals, we performed measurement on blood routine, biochemical indices, and inflammatory cytokines. We found that hemoglobin concentration, mean corpuscular hemoglobin concentration, red blood cell count, high-density lipoprotein (HDL), total bile acid, total protein, albumin, apolipoprotein A1, blood urea nitrogen, and kalium were lower in the case group than in the healthy controls (Figures 1A–C,F–L, respectively; *p* < 0.05). Meanwhile, WBC count, neutrophil count, and natrium were higher in the case group than in the healthy controls (Figures 1D–F,M, respectively; *p* < 0.05). The remaining 20 parameters did not significantly differ between the case group and healthy controls (Supplementary Table 1; *p* > 0.05). We continued our analysis in SCZ patients and healthy controls to determine whether dysbiosis of inflammatory cytokines existed in the two groups. Results showed that the plasma levels of CRP, IL-8, IL-16, IL-6, IL-13, IL-10, and IL-15 significantly increased in the case group than in the healthy controls (Figures 1N–R,T,U, respectively; *p* < 0.05), whereas IL-7 levels were significantly lower than in the healthy controls (Supplementary Figure 1; *p* < 0.05). Conversely, no significant differences were found in terms of the Flt-1, PIGF, Tie-2, IFN-γ, TNF-α, TNF-β, IL-1α, IL-2, IL-5, IL-12, and IL-17A between the two groups (Supplementary Table 1; *p* > 0.05). More details are shown in Supplementary Material.

### Correlations of Blood Routine, Biochemical, and Cytokine Levels With Psychotic Symptom

**Figure 2:**
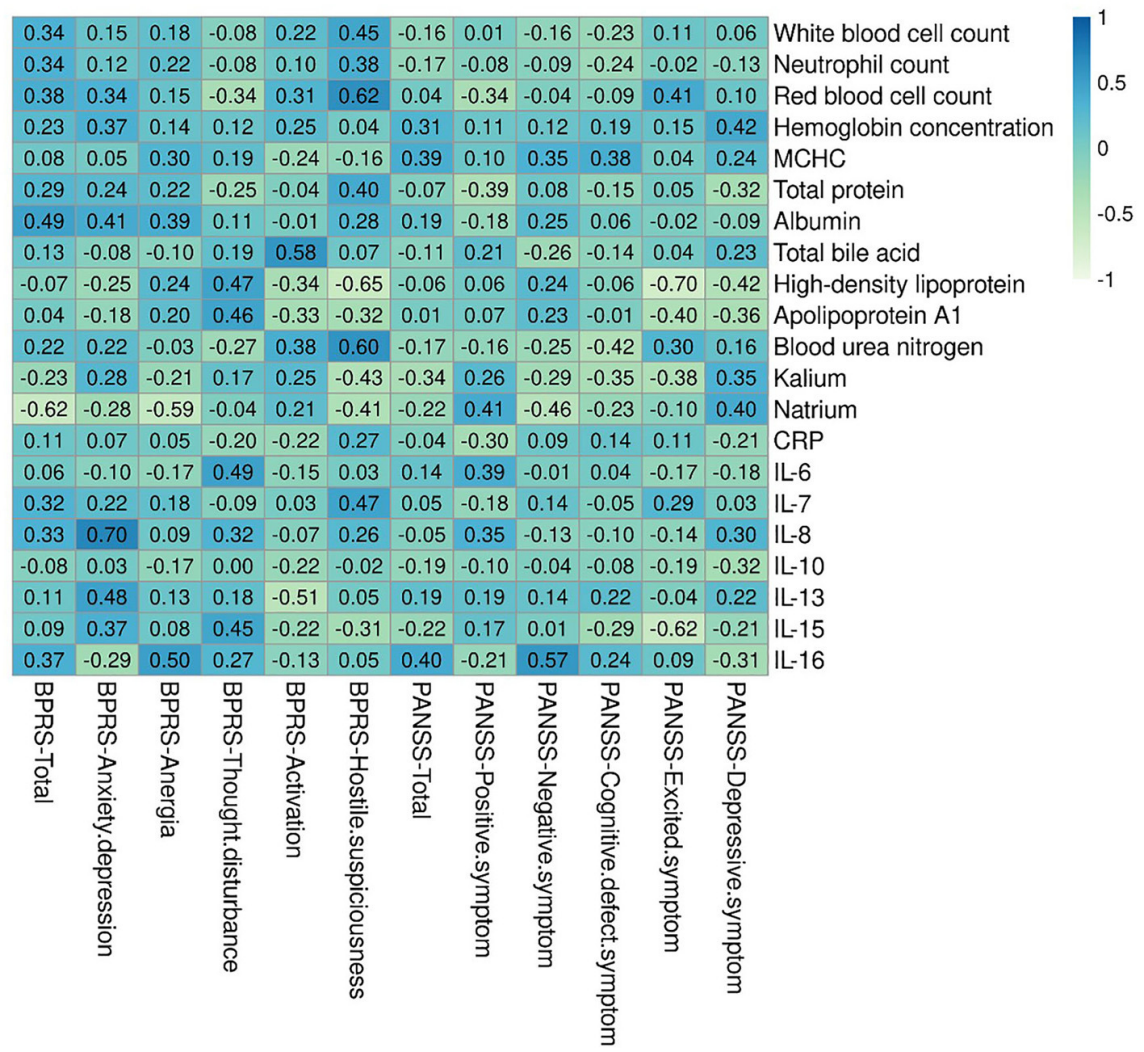
Correlations heatmap of blood routine, biochemical, and inflammation cytokines with clinical symptom. There are six indexes had significantly positively and negatively connection with clinical indicators of BPRS, including red blood cell count, blood urea nitrogen, total bile acid, IL-8, Natrium, and High-density lipoprotein. There are three indexes had significantly positively and negatively connection with clinical indicators of PANSS. BPRS, Brief Psychiatric Rating Scale; PANSS, Positive and Negative Syndrome Scale.

Figure 2 shows the correlations of blood routine, biochemical, and inflammation cytokines with clinical symptom in patients with SCZ. Four indices were revealed to have a significant positive correlation with BPRS, including red blood cell count and blood urea nitrogen with hostile and suspiciousness subscale (*r* = 0.622, *p* = 0.013, and *r* = 0.598, *p* = 0.018, respectively), total bile acid with activation subscale (*r* = 0.579, *p* = 0.024), and IL-8 with anxiety-depression subscale (*r* = 0.704, *p* = 0.003). Contrary to these positive correlations, two indices showed mostly negative correlation with clinical indicators of BPRS, namely, HDL with hostile and suspiciousness subscale (*r* = −0.65, *p* = 0.009), natrium with anergia subscale, and total scores (*r* = −0.59, *p* = 0.038; *r* = −0.62, *p* = 0.013). Apart from BPRS, we also revealed one index that had significant positive correlation with clinical indicators of PANSS, i.e., IL-16, with negative symptom subscale (*r* = 0.569, *p* = 0.027). Regarding the negative correlation prediction with clinical indicators of PANSS, two indices were found, including HDL and IL-15, with excited symptom subscale (*r* = −0.705, *p* = 0.003; *r* = −0.617, *p* = 0.014).

### Correlation Network of Blood Routine, Biochemical Indices, and Inflammatory Cytokine Levels With Clinical Symptoms

**Figure 3:**
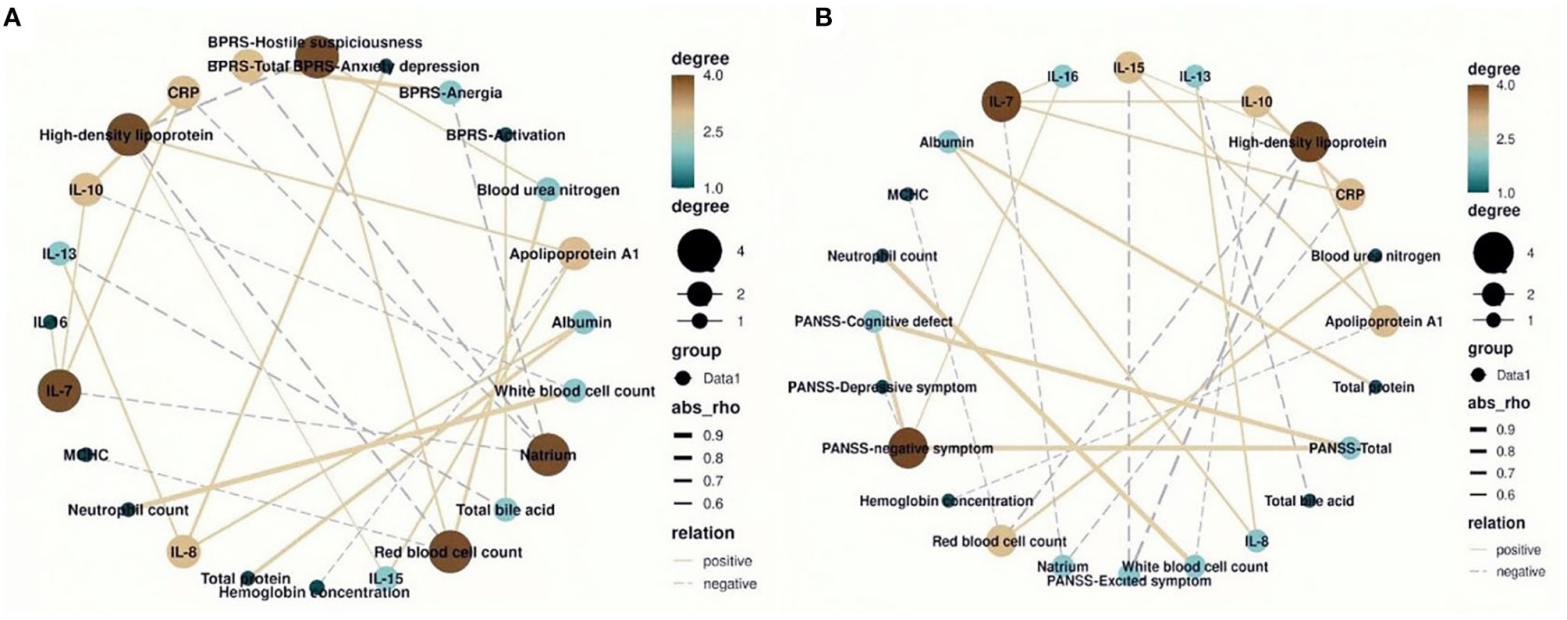
The correlation network between blood routine, biochemical, and inflammatory factors and total and subscale scores of Brief Psychiatric Rating Scale (BPRS) and Positive and Negative Syndrome Scale (PANSS). **(A)** The natrium and red blood cell count had the highest degree of correlation with the BPRS (degree = 4). **(B)** The high-density lipoprotein had the highest degree of correlation with the PANSS (degree = 4).

Results showed that the metabolic biochemical indices of natrium, HDL, and red blood cell count were most closely correlated with BPRS-related clinical symptoms among the correlation network (Figure 3A; degree = 4). In particular, natrium was negatively correlated with the total scores of BPRS (Figure 3A; *r* = −0.622, *p* = 0.013) and anergia symptom subscale (Figure 3A; *r* = −0.589, *p* = 0.021). Furthermore, we found that HDL was negatively correlated with the hostile suspiciousness symptom subscale and red blood cell count and was simultaneously positively correlated with IL-15 (Figure 3A; *r* = −0.645, *p* = 0.009; *r* = −0.615, *p* = 0.016; *r* = −0.535, *p* = 0.046, respectively). We also observed that IL-8 was positively correlated with clinical symptoms (Figure 3A; *r* = 0.701 *p* = 0.003) and also positively correlated with albumin and IL-13 (Figure 3A; *r* = 0.651 *p* = 0.006; *r* = 0.624 *p* = 0.011, respectively). As expected, HDL had the highest degree of correlation with PANSS-related clinical symptoms in the correlation network (Figure 3B; degree = 4), similar to the finding on BPRS, in which HDL was found to be negatively correlated with excited symptom score subscale and red blood cell count (Figure 3B; *r* = −0.705, *p* = 0.003; *r* = −0.611, *p* = 0.016). Interestingly, results of the correlation network revealed that the negative symptoms were the core symptoms of this disease, which directly affected the cognitive function symptoms and PANSS total scores (Figure 3B; *r* = 0.821, *p* < 0.0001; *r* = 0.781, *p* < 0.0001, respectively). Furthermore, we found that IL-16 had positive correlation with the negative symptoms (Figure 3B; *r* = 0.571, *p* = 0.027).

### Predictive Analysis of Validity and Effectiveness of These Aberrant Parameters SCZ Progression

**Figure 4:**
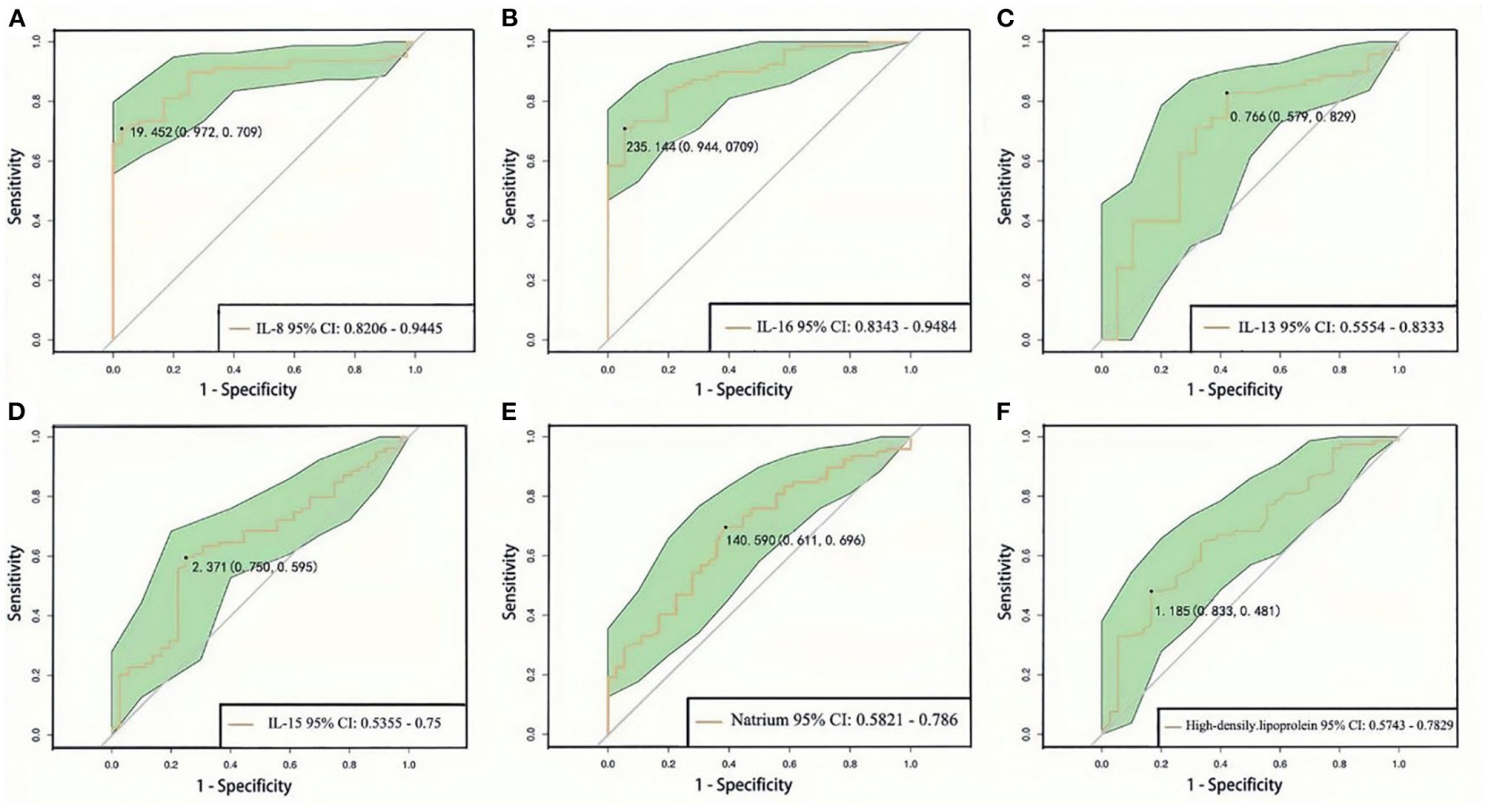
ROC curve analysis of blood routine, biochemical, and inflammation cytokines between first episode drug-naïve schizophrenia patients and healthy controls **(A–F)**. 95% CI, 95% confidence interval.

ROC curve analysis was used to predict the progression of SCZ clinical symptoms by using dysbiosis of blood routine, biochemical indices, and inflammatory cytokines. We found that IL-8 can predict the progression of SCZ at a cutoff level of 19.452 pg/L, with a specificity of 0.972 and sensitivity of 0.709 (Figure 4A). IL-16 can predict SCZ progression at a cutoff level of 235.144 pg/L, with a specificity of 0.944 and sensitivity of 0.709 (Figure 4B). IL-13 can predict SCZ progression at a cutoff level of 0.766 μg/L, with a specificity of 0.579, and sensitivity of 0.829 (Figure 4C). IL-15 can predict SCZ progression at a cutoff level of 2.371 pg/L, with a specificity of 0.750 and sensitivity of 0.595 (Figure 4D). Natrium can predict SCZ progression at a cutoff level of 140.590 mmol/L, with a specificity of 0.611 and sensitivity of 0.696 (Figure 4E), HDL can predict SCZ progression at a cutoff level of 1.185 mmol/L, with a specificity of 0.833 and sensitivity of 0.481 (Figure 4F). Predictive analysis results revealed that the dysbiosis parameters of IL-8, IL-16, IL-13, IL-15, natrium, and HDL were associated with predicting prognostic risk in SCZ progression.

## Discussion

The development of SCZ may be driven by interactions with multiple vulnerability factors, which culminate in the expression of disease state and indices. In this study, we aimed to examine the association of a large comprehensive panel of inflammatory markers and various plasma biochemical indicators with SCZ clinical symptomatology. We found that differential values of blood routine, biochemical indices, and inflammatory cytokine levels between two groups. These risk factors may be cumulative and interactive with each other and with critical periods of neurodevelopmental vulnerability. Thus, understanding the complex interactions among these influences in the pathogenesis of SCZ is highly important. We found six indices that were positively and negatively correlated with clinical indicators of BPRS and three indices that were positively and negatively correlated with clinical indicators of PANSS. The complex interactions between inflammatory responses and metabolic dysfunction may have etiological significance for SCZ. Results of correlation-network analysis showed that natrium, HDL, IL-8, IL-16, and IL-15 were the most closely correlated with clinical symptoms. ROC analysis was conducted and yielded six indices with high specificity and sensitivity to predict the association of the progression of SCZ clinical symptoms with dysbiosis of blood routine, biochemical indices, and inflammatory cytokines.

Several studies have shown that homeostasis state plays a pivotal role in SCZ pathogenesis. SCZ progression was found to be associated with metabolic disease morbidity and mortality, including aberrant levels of blood routine, biochemical indices, and inflammatory cytokines. In line with previous research, we found that WBC count and neutrophil count were higher in the case group than in the healthy controls, whereas red blood cell count, hemoglobin concentration, and HCMC were lower than in the healthy controls (25, 26). We also observed a somewhat discordant pattern of biochemical results between two groups, i.e., natrium was higher, whereas total protein, albumin, total bile acid, HDL, apolipoprotein A1, blood urea nitrogen, and kalium were lower in the case group (27, 28). Furthermore, we found that the plasma levels of CRP, IL-8, IL-6, IL-10, IL-13, and IL-16 significantly increased in the case group than in the healthy controls, whereas the plasma level of IL-7 decreased. These results indicated the need to consider the state of low-grade elevation inflammation and nutritional deficiency in SCZ patients. In the future, we will carry out a multilevel evaluation of patients and establish a novel approach for the detection, treatment, and prevention of psychotic disorders.

Additionally, we detected the relationship of dysbiosis of blood routine and biochemical factors with SCZ clinical symptoms. Results showed that red blood cell count, total bile acid, and blood urea nitrogen had positive correlation with clinical symptom of BPRS, whereas HDL and natrium had negative correlation with clinical symptoms of BPRS and PANSS. Red blood cells are the common type of blood cell, and their important function is the transport of oxygen. The correlation between red blood cells' low-level structure and SCZ has long been recognized, consistent with our findings. The most common cause of such abnormality may be limited activity, poor economic conditions, and abnormal eating factors (29). A nutritional-deficiency state has a risk susceptibility linked to possible metabolic disorders in SCZ patients (30). Misiak et al. found a number of metabolic abnormalities related to impaired glucose metabolism, low levels of HDL, and high levels of triglycerides and hsCRP in drug-naïve SCZ patients (31). Gjerde et al. found an increase in HDL level associated with an improvement in negative symptoms and cognitive performance in first-episode psychosis after 1 year of antipsychotic treatment (32). These findings warrant further investigation and should be considered in relation to the interaction between lipid pathways and psychosis. Furthermore, we found that IL-8 and IL-16 had positive correlation with clinical symptoms of BPRS and PANSS, whereas IL-15 had negative correlation with clinical symptom of PANSS. These results were consistent with numerous studies reporting the elevated plasma concentrations of pro-inflammatory cytokines and descending anti-inflammatory cytokines are associated with severe SCZ clinical symptoms (33, 34). Thus, inflammatory cytokines may be involved in the susceptibility to SCZ, but further independent analyses are needed.

SCZ is a highly heterogeneous disease, so the development of SCZ may be driven by interactions with multiple vulnerability factors. Accordingly, we constructed a correlation network to depict the co-occurrence interaction between the dysbiosis parameters correlated with SCZ clinical symptoms. Ideally, through the connectivity and relationships of these networks, we can elucidate the authentic internal state of SCZ patients. Herein, the dysbiosis of natrium, HDL, IL-8, IL-16, and IL-15 were found to be the most closely associated with BPRS- and PANSS-related excitement, suspiciousness, and negative symptoms. The interplay between dyslipidemia of HDL, chronic inflammation, and psychotic symptoms suggested a strong impact of inflammatory dysregulation on metabolic risk in these patients. In particular, we clarified the potential of dysbiosis peripheral inflammatory markers as predictors of clinical outcomes in patients with psychosis. The main inflammatory markers associated with clinical outcome in psychosis were IL-15, IL-13, IL-16, and IL-8, which may be associated with poor clinical outcome. The effectiveness and validity of these aberrant parameters as predictors of SCZ symptoms need to be determined to enable the development of more targeted predictors. The mechanisms through which peripheral inflammation could contribute to poor clinical outcome are still partly unclear (35–37). Consistently, several studies have demonstrated that the IL-15, IL-16, and IL-8 levels in SCZ patients are associated with a poor response to antipsychotic treatment, and increased IL-8 levels are significantly associated with more severe negative symptoms in patients. These findings suggest that these pro-inflammatory cytokines are potential markers to identify patients with poor clinical outcome (38). Interestingly, other studies have reported that IL-13 may be involved in the improvement of psychopathology and symptomatologies in SCZ, suggesting that an anti-inflammatory response as mediated by the type-2 T helper cell pathway may contribute to a treatment-induced recovery in psychosis. Future studies need to consider the possible balance between pro- and anti-inflammatory pathways and how they contribute to clinical outcome (39).

Complex dysfunction of genetic and heterogeneous environment factors had demonstrated to comprise the key etiological features intrinsic to SCZ, and beyond the role of blood routine, biochemistry and inflammation factors we had discussed above, the demographic characteristic factors should be taken into account to study comprehensive about its etiology and pathological mechanism in the course and prognostic importance of patients with SCZ. More recently, investigators proved sex differences also influence the illness onset, clinical presentations and treatment response in the majority of psychiatric disorders, with women with SCZ are at higher risk for increased inflammatory marker levels compared to men, possibly related to the immunomodulatory function of estrogen (40, 41). Therefore, subsequent research should explore the gender-specific factors with the pathogenesis of SCZ. It is noteworthy to mention that knowledge about an array of risk factors for SCZ, the duration of untreated psychotic (DUP) is an important factor for patients and is generally considered to be the period from onset of psychotic symptoms to the initiation of treatment, as well as a longer DUP had been considered leading to poorer outcomes, however, the mechanisms linking longer DUP to poorer clinical outcomes are still unclear (42, 43). It is unknown during the DUP stage that the factors of inflammatory and biochemistry which to be ascribed to environmental influences whether participate in the disease process of SCZ. Based on the previous research, the synergy of environment is still an important factor for us to consider the etiology of SCZ. This study has several strengths. First, was the use of more homogeneous first-episode drug-naïve SCZ patients with normal weight. In this way, we minimized the potential confounding factors of antipsychotic drugs and obesity on the levels of biochemical index, blood routine, and inflammatory cytokines. Second, we utilized the MSD platform to examined 20 inflammatory factors simultaneously. This multiplex platform has good reliability and can detect comprehensive biomarkers simultaneously. The present study also has several noteworthy limitations requiring further investigation. First, our design was a cross-sectional, and future studies should include analyses on the correlation of longitudinal biomarkers analyses with disease progression. Second, the sample size in this clinical research was relatively small; thus, future studies should target larger-sized cohorts of SCZ. Third, this is a female-dominated group, and the average illness onset is relatively high, which might have impacts on the results and gender-specific studies are warranted in future research. Fourth, the DUP may influence multiple indicators of onset symptoms, metabolic syndrome, and disease prognosis in patients with first-episode SCZ, a factor will be further investigated in our future studies. Despite of these limitations, this work provided a new perspective on the immune dysregulation in SCZ to some extent.

## Conclusion

Our findings revealed the interactive networks of biological immunity profiles and psychotic symptoms. HDL, natrium, red cell count, and IL-8 played a core role in the network model of correlation. HDL, IL-16, IL-8, and natrium could partly explain the variance in symptom score of SCZ patients. All our findings provided new insights to elucidate the assessment between subtypes of SCZ and potentially enable the development of more effective and targeted therapeutics in patients with SCZ spectrum disorders.

## Data Availability Statement

The raw data supporting the conclusions of this article will be made available by the authors, without undue reservation.

## Ethics Statement

The studies involving human participants were reviewed and approved by the Medical Ethics Committee of the Anhui Mental Health Center. The patients/participants provided their written informed consent to participate in this study.

## Author Contributions

CZ, XZ, and QX were responsible for the study design and manuscript editing. JY, YC, and PJ were responsible for literature searches, statistical analyses, and manuscript writing. JL, KW, JZ, CL, LZ, and JG were responsible for clinical-scale assessment data collection. All authors contributed to the article and approved the submitted version.

## Funding

This study was supported by funding from the Loan-to-Replenishment Project in Hefei, Anhui Province, China (grant number J2018Y05), the Hospital Project of Hefei Fourth People's Hospital (grant number 2019023), the Fund Project of Anhui Medical University (grant number 2019xkj206), the Shanghai Key Laboratory of Psychotic Disorders Open Grant (grant number 13dz2260500), the Natural Science Research Projects in Anhui Universities (grant number KJ2020A0218), the Applied Medicine Research Project of Hefei Health Committee (grant number Hwk2020zd0016), and the Scientific and Technological Research Project of Anhui Provincial Science and Technology Department (201904a07020009). The funding sources had no involvement in study design, collection, analysis, writing of this paper, and publication.

## Conflict of Interest

The authors declare that the research was conducted in the absence of any commercial or financial relationships that could be construed as a potential conflict of interest.

## Publisher's Note

All claims expressed in this article are solely those of the authors and do not necessarily represent those of their affiliated organizations, or those of the publisher, the editors and the reviewers. Any product that may be evaluated in this article, or claim that may be made by its manufacturer, is not guaranteed or endorsed by the publisher.
