# Supplementary material for: Network Association of Biochemical and Inflammatory Abnormalities With Psychiatric Symptoms in First-Episode Schizophrenia Patients
Source: Front Psychiatry. 2022 Feb 22;13:834539. doi: 10.3389/fpsyt.2022.834539 (PMC8901486; doi:10.3389/fpsyt.2022.834539)
Supplement: Supplementary file 1 [file Table_1.DOCX]

| **Table S1 Comparation of concentrations of immunobiological profiles between healthy controls (HC) and first episode drug naïve patients with schizophrenia (SCZ)** | | | | |
| --- | --- | --- | --- | --- |
| **Variables** | **Normal range** | **HC** | **SCZ** | **t/Z (*P)*** |
| Lymphocyte count (×10^9^/L) | 1.10-3.20 | 1.96±0.54 | 1.84±0.59 | 1.097 (0.275) |
| Monocyte count (×10^9^/L) | 0.10-0.60 | 0.37±0.15 | 0.38±0.13 | -0.528 (0.599) |
| Eosinophil count (×10^9^/L) | 0.02-0.52 | 0.09±0.06 | 0.12±0.11 | -1.510 (0.134) |
| Basophil count (×10^9^/L) | 0.02-0.52 | 0.02±0.01 | 0.03±0.02 | -1.353 (0.179) |
| Platelet count (×10^9^/L) | 125-350 | 219.75±50.59 | 203.50±56.45 | 1.477 (0.142) |
| Total bilirubin (μmol/L) | 0-17.1 | 12.60±5.54 | 12.26±6.86 | 0.262 (0.794) |
| Direct bilirubin (μmol/L) | 0-6.3 | 4.05 (3.43, 5.40) | 4.00 (3.20, 5.50) | -0.175 (0.861) |
| Globulin (g/L) | 20.00-40.00 | 25.29±3.55 | 24.13±3.61 | 1.601 (0.112) |
| Alanine transaminase (U/L) | 7-45 | 14.50 (10.25, 28.25) | 15.90 (11.00, 27.00) | -0.420 (0.675) |
| Aspartate aminotransferase (U/L) | 13-40 | 16.50 (14.00, 23.00) | 17.00 (15.00, 24.00) | -0.871 (0.384) |
| Alkaline phosphatase (IU/L) | 35-100 | 65.69±21.01 | 65.93±18.64 | -0.059 (0.953) |
| Gamma-glutamyltransferase (IU/L) | 7-45 | 18.50 (12.00, 29.00) | 15.00 (11.00, 24.00) | -1.211 (0.226) |
| Total cholesterol (mmol/L) | 2.50-5.20 | 4.18±0.90 | 3.92±0.78 | 1.605 (0.111) |
| Triglyceride (mmol/L) | 0.34-2.28 | 1.09±0.66 | 1.29±0.81 | -1.300 (0.196) |
| Apolipoprotein B (g/L) | 0.60-1.19 | 0.83±0.80 | 0.73±0.19 | 1.098 (0.274) |
| Uric acid (μmol/L) | 155.00-428.00 | 346.32±130.87 | 330.78±90.57 | 0.738 (0.462) |
| Chlorine (mmol/L) | 99.00-110.00 | 102.87±17.36 | 105.20±3.04 | -1.158 (0.249) |
| Creatine kinase (U/L) | 26-174 | 84.50 (61.00, 115.50) | 92.00 (58.00, 166.00) | -1.149 (0.251) |
| Glucose (mmol/L) | 3.85-6.11 | 5.27±1.18 | 4.92±0.93 | 1.697 (0.092) |
| Magnesium (mmol/L) | 0.66-1.05 | 0.87±0.06 | 0.89±0.10 | -0.718 (0.474) |
| Flt-1 (pg/ml) | - | 111.30±24.62 | 107.37±34.76 | 0.612 (0.542) |
| PIGF (pg/ml) | - | 8.10 (6.96, 9.81) | 8.42 (7.15, 10.41) | -0.953 (0.341) |
| Tie-2 (pg/ml) | - | 2222.74±520.69 | 2053.62±628.92 | 1.408 (0.162) |
| IFN-γ (pg/ml) | - | 7.27 (4.31, 10.31) | 7.47 (4.19, 11.40) | -0.217 (0.828) |
| TNF-β (pg/ml) | - | 0.12 (0.08, 0.17) | 0.13 (0.10, 0.19) | -1.077 (0.282) |
| IL-1α(pg/ml) | - | 0.29 (0.22, 0.62) | 0.29 (0.14, 0.58) | 0.159 (0.873) |
| IL-2 (pg/ml) | - | 0.21 (0.08, 0.37) | 0.24 (0.15, 0.38) | 1.099 (0.272) |
| IL-4 (pg/ml) | - | 0.0075 (0.0031, 0.0205) | 0.0128 (0.0073, 0.0281) | -1.946 (0.052) |
| IL-5 (pg/ml) | - | 0.13 (0.04, 0.34) | 0.18 (0.10, 0.35) | 1.327 (0.185) |
| IL-12 (pg/ml) | - | 104.79±45.85 | 108.56±54.85 | -0.358 (0.721) |
| IL-17A (pg/ml) | - | 2.53±1.43 | 3.12±2.50 | -1.328 (0.187) |
| CRP, C-reactive protein; IL-, interleukin-; IFN-γ, Interferon gamma; PIGF, Placental growth factor; TNF-, tumor necrosis factor-; | | | | |
